# Dosimetric Feasibility of Hypofractionated Radiation Therapy with Simultaneously Integrated Central Boost for Retroperitoneal Sarcoma

**DOI:** 10.3390/cancers18193114

**Published:** 2026-09-25

**Authors:** Zoe L. Cosner, Katie Lee, Yaguang Pei, Kevin X. Liu, Elizabeth H. Baldini, Miranda B. Lam

**Affiliations:** 1 Department of Radiation Oncology, Brigham and Women’s Hospital and Dana-Farber Cancer Institute, Boston, MA 02115, USA; 2 Department of Radiation Oncology, Massachusetts General Hospital, Boston, MA 02114, USA; 3 Department of Radiation Oncology, Mayo Clinic, Rochester, MN 55905, USA

**Keywords:** cancer, hypofractionated radiation therapy, retroperitoneal sarcoma, simultaneous integrated boost

## Abstract

**Background**: Retroperitoneal sarcomas (RPS) are rare malignancies that pose challenges due to their large size and proximity to organs-at-risk (OARs). Compared to conventional 5-week radiation therapy (RT), hypofractionated RT (HFRT) offers a shorter alternative with potential biologic benefit, and simultaneous integrated boost (SIB) can increase delivered dose. We evaluated the dosimetric feasibility of HFRT with a central tumor SIB for bulky RPS. **Methods**: Fifteen patients with RPS previously treated with preoperative conventionally fractionated RT were replanned using HFRT with a central SIB. Volumetric modulated arc therapy (VMAT) plans were created to deliver 42.75 Gy in 15 fractions to the planning target volume (PTV), 45 Gy to the internal gross tumor volume (iGTV), and 52.5 Gy to the SIB volume (1 cm contraction of iGTV). OAR planning objectives were prioritized during optimization. Dosimetric plan-generation feasibility was defined as generation of a treatment plan meeting prespecified target-coverage criteria; compliance with each OAR objective was assessed separately. **Results**: Mean tumor size was 18.3 cm (range 5.8–28.2 cm), with mean volumes for iGTV, SIB, and PTV of 1633.8 cc, 926.9 cc, and 3323.2 cc, respectively. All plans met the prespecified target coverage criteria for iGTV, SIB, and PTV. Ten of 15 plans met all target-coverage criteria and all prespecified OAR objectives; five met the target coverage criteria and non-bowel OAR objectives but exceeded at least one bowel objective by <1% of the specified constraint. All non-bowel OAR objectives were met except that the bilateral kidney objective was exceeded in 4 of 11 evaluable patients. The average homogeneity index for the iGTV and SIB were 0.21 (range 0.18–0.25) and 0.05 (range 0.04–0.06), respectively. The PTV 95% conformality index was 0.99 (range 0.97–1.0), indicating excellent target conformation with minimal normal tissue spillage. **Conclusions**: These findings support the dosimetric feasibility of generating hypofractionated radiation therapy plans with a central SIB for large retroperitoneal sarcomas. However, one-third of plans exceeded at least one bowel objective, and these planning results do not establish clinical deliverability, safety, or efficacy.

## Simple Summary

Retroperitoneal sarcomas are often very large and located close to sensitive organs, making radiation therapy challenging. This study evaluated whether a shorter, hypofractionated radiation therapy schedule with a simultaneous integrated boost to the center of the tumor could be planned for bulky tumors while limiting dose to nearby organs. Treatment plans for 15 patients were successfully created, delivering 42.75 Gy to the planning target, 45 Gy to the internal tumor volume, and 52.5 Gy to the central boost volume over 15 treatments. All plans met prespecified target-coverage criteria. Non-bowel objectives were met except that the bilateral kidney objective was exceeded in 4 of 11 evaluable patients. Five patients exceeded at least one bowel objective by less than 1% of the specified value. These findings support the dosimetric plan-generation feasibility of hypofractionated radiation therapy with a central tumor boost for bulky retroperitoneal sarcoma but do not establish the clinical deliverability, safety, or efficacy of the regimen.

## 1. Introduction

Retroperitoneal sarcomas (RPS) are rare malignancies, comprising 1–2% of solid malignancies and 10–20% of sarcomas [1]. They present significant therapeutic challenges due to their size, anatomical location, and tendency for local recurrence [2]. The current standard of care is complete macroscopic resection [3], which offers the best chance for long-term control [3]. However, given the complex anatomy of the retroperitoneum with critical structures and the large presenting size, some tumors are deemed to be inoperable [4]. Further, for patients who have had recurrent disease, re-operation can be challenging, and alternative approaches for local control are desired [5].

Conventional preoperative RT, typically delivered as 50–50.4 Gy in 1.8–2 Gy fractions over five weeks, has been explored in both retrospective review and prospective trials, but its role remains controversial due to mixed outcomes and concerns about toxicity to adjacent organs at risk (OARs) [6,7,8]. In response, newer strategies are being investigated to enhance RT efficacy and minimize morbidity. One such approach is dose escalation to regions deemed at high risk for positive resection margins using a simultaneous integrated boost (SIB) technique [9,10,11]. This method aims to intensify the radiation dose to subregions within the tumor while sparing surrounding normal tissues. For unresectable or bulky tumors, a central boost SIB has been used in the standard fractionation paradigm to obtain local control and prolong time to systemic therapy [12,13].

Hypofractionated RT (HFRT) has emerged as a promising alternative to conventional fractionation [14]. HFRT may take advantage of the potentially low α/β ratio of soft tissue sarcomas, theoretically increasing the biologically effective dose (BED) to the tumor without proportionally increasing late-reacting OAR toxicity [15]. Significantly, HFRT reduces the treatment burden for patients, decreasing the number of hospital visits and associated logistical and financial stresses [16]. An innovative evolution of this strategy is the use of HFRT to the entire tumor volume, combined with a central tumor SIB in unresectable or bulky tumors. By avoiding the delivery of the highest doses to the tumor-OAR interface, this technique may mitigate the risk of severe toxicities such as bowel perforation. Tumor necrosis and immunomodulatory effects have been proposed as potential biological mechanisms supporting central tumor dose escalation, although their relevance to this regimen and disease setting remains unknown [17]. This strategy has been used clinically in a small number of patients (Table S1) [18,19,20].

In this study, we investigate the dosimetric feasibility of HFRT with a central tumor SIB in patients with bulky RPS located throughout the abdomen and pelvis. To rigorously test the approach, we deliberately selected tumors with extensive involvement across all areas of the retroperitoneum. We hypothesize that it will be feasible to create dosimetry plans for bulky RPS using a 3-week HFRT to whole tumor with central tumor SIB while maintaining excellent target coverage and maintaining normal organ constraints.

## 2. Materials and Methods

### 2.1. Patient Selection and Simulation

Fifteen patients with RPS treated at our institution with conventional pre-operative 50 Gy radiation therapy between 2017 and 2024 who were enrolled as part of a prospective biobanking study were included for replanning with hypofractionated radiation therapy (HFRT) with central simultaneous integrated boost (SIB). Eligible patients were over the age of 18 with bulky RPS tumors. This study was approved by the Institutional Review Board with a full waiver of informed consent. CT simulations from patients’ initial treatment course were used for target definition and dosimetric planning. Each patient underwent four-dimensional CT (4D-CT) with oral contrast. These patients initially underwent surgical resection and had tumors with significant tumor burden (mean diameter 18.3 cm) and complex anatomical locations, which may have been considered unresectable at other institutions. This cohort allows for a rigorous test of the HFRT-SIB technique in cases where bulky disease poses the greatest risk of OAR encroachment.

### 2.2. Target and Organ at Risk (OAR) Definition

To account for respiratory motion, an internal gross tumor volume (iGTV) was contoured on the Average Intensity Projection Scan. The iGTV was expanded by 1.5 cm and then edited per expert consensus guidelines to form the CTV [21]. The CTV was isotropically enlarged by 5 mm to form a PTV. The SIB volume was created by isotropically contracting the iGTV by 1 cm (Figure 1). OARs were contoured, including the spinal canal, duodenum, large bowel, small bowel, bowel bag, rectum, kidneys, lungs, hearts, bladder, liver, femoral heads, and anus/vulva/perineum as appropriate. Small bowel, large bowel, and duodenum contours did not overlap.

### 2.3. Dose Planning and Plan Evaluation

Volumetric arc therapy (VMAT) and sliding window intensity-modulated radiation therapy (IMRT) plans were generated on Eclipse treatment planning system 18.0 (Varian Medical Systems, Palo Alto, CA, USA). Treatment plans were initially generated using VMAT, with IMRT employed when dose constraints could not be adequately achieved. All plans were optimized using photon optimizer version 18.0.1 and calculated on a 2 mm grid size using the anisotropic analytical algorithm version 18.0.1. All plans were designed to be delivered by a Varian TrueBeam linear accelerator. The OAR metrics were prioritized over prescription coverage (Table 1). OAR constraints for the hypofractionated approach were selected based on national and international guidelines, discussion with sarcoma and gastrointestinal radiation oncology experts, and Equivalent Dose in 2 Gy fractions (EQD2) calculations [14] (Table S5). The treatment course was planned for 15 fractions. The prescription doses for PTV, iGTV, and SIB were 42.75 Gy, 45 Gy, and 52.5 Gy, respectively (Table 2). A 1 cm isotropic contraction was selected pragmatically to separate the central boost from the tumor-OAR interface and provide space for dose falloff; it was a planning choice rather than a biologically validated margin. Target prescription metrics were ≥95% of PTV at 95% of prescription dose and ≥95% of iGTV and SIB at 98% of prescription dose. Plans were evaluated for ability to meet OAR metrics, prescription dose coverage, dose homogeneity, and conformity. Dose homogeneity was calculated using a homogeneity index (HI) with the formula HI = (D_5%_ − D_95%_)/D_p_. Dose conformity was calculated using the conformity index (CI) with the formulas CI_100%_ = V_100%PTV_/TV_PTV_, and CI_95%_ = V_95%PTV_/TV_PTV_ (TV = total volume). The prespecified dosimetric plan-generation feasibility endpoint was generation of a treatment plan meeting target-coverage criteria while optimizing toward the OAR objectives in Table 1. Compliance with each OAR objective was assessed and reported separately. The magnitude of a deviation did not retrospectively reclassify that objective as met.

## 3. Results

### 3.1. Study Population and Statistical Analysis

Fifteen patients with RPS previously treated with conventional pre-operative 50 Gy radiation therapy were included (Table 3). Ten (66.7%) patients were female. Histologies included 4 (26.7%) well-differentiated liposarcoma, 8 (53.3%) dedifferentiated liposarcoma, 1 (6.7%) undifferentiated pleomorphic sarcoma, 1 (6.7%) pleomorphic spindle cell sarcoma, and 1 (6.7%) myxoid and spindle cell sarcoma. The majority of tumors were located in the retroperitoneum, with 1 (6.7%) tumor located in the pelvis. The mean size of tumors was 18.3 cm (range 5.8–28.2 cm). Eight (53.3%) were right-sided, and 7 (46.7%) were left-sided. Three (20%) tumors were recurrent, while the rest were primary tumors (80%). The majority of patients had an ipsilateral nephrectomy associated with treatment; 2 (13.3%) patients had nephrectomy prior to the time of CT simulation, 11 (73.3%) had nephrectomy at the time of definitive surgery post-RT, 1 (6.7%) had partial nephrectomy after RT, and 1 (6.7%) patient had their post-RT tumor resection canceled and thus did not have a nephrectomy, though it had originally been planned. Our analyses for this paper were descriptive.

### 3.2. Evaluation of OAR Metrics

Eleven plans were generated using VMAT, with 4 plans requiring sliding window IMRT planning to meet constraints. All patients had reported large bowel, small bowel, duodenum, liver, contralateral kidney, and spinal cord doses; however, not all patients had reported doses for bilateral kidney and femoral head. Due to certain tumor locations, the complete femoral head anatomy was not always included within the CT simulation field of view. Bladder metrics were not included in the aggregate analysis because the complete bladder was not consistently captured within the simulation field of view. In regard to bilateral kidney volumes, two patients had only one kidney at the time of CT simulation, and two patients had an ipsilateral kidney that was not discernible from the tumor volume. In four patients who had two kidneys, but whose ipsilateral kidney was abutted or was encased by tumor, bilateral kidney constraints were recorded; however, only the contralateral kidney constraint was used for treatment plan evaluation, as per protocol. Across all patients, all non-bowel OAR constraints were met except for bilateral kidneys in four patients. Of 15 plans, 10 met all target-coverage criteria and all prespecified OAR objectives. The remaining five met target-coverage criteria and non-bowel OAR objectives but exceeded at least one bowel objective by less than 1% of the specified value (Figure 2). For the Dmax bowel constraint, both D0.03 and point max were obtained. (Tables S2–S4).

Among the five plans with a bowel-objective deviation, four involved the large bowel only, and one involved both the small bowel and large bowel. The duodenal constraint was met in all cases. The largest excess bowel dose was a large bowel maximum dose of 45.4 Gy (constraint ≤ 45 Gy); this patient also had a large bowel V42.75 of 50.1 cc (constraint ≤ 50 cc). For three of the patients, the only constraint exceeded was the maximum dose, with constraints for V42.75 and D0.03 met in each case. For the patient who exceeded both small and large bowel constraints, both were a maximum dose of 45.1 Gy (constraint ≤ 45 Gy for both), with V42.75 and D0.03 constraints met. The patients in which constraints were not strictly met were in cases where bowel abutted tumor volumes.

### 3.3. Evaluation of Target Coverage

The mean treatment volumes for iGTV, SIB, and PTV were 1633.8 cc (range, 188.53–8894.5 cc; median [IQR], 1256.2 [390.4–1623.5] cc), 926.9 cc (range, 18.6–6664 cc; median [IQR], 498.6 [97.6–879.6] cc), and 3323.2 cc (range, 567.2–12989.3 cc; median [IQR], 3095.6 [1162.9–3833.55] cc), respectively. All plans met the coverage metrics for iGTV, SIB, and PTV while prioritizing the OAR constraints (Figure 3A). Maximum SIB dose of 110% was also met in all patients. The average HI of the iGTV and SIB were 0.21 (range 0.18–0.25) and 0.05 (range 0.04–0.06), respectively (Figure 3B). These HI are indicative of relatively good homogeneity, with the higher homogeneity in the iGTV related to dose fall-off from the SIB. The PTV CI_95%_ average was 0.99 (range 0.97–1.0), and the PTV CI_100%_ average was 0.91 (range 0.81–0.97), which are consistent with good isodose-to-treatment-volume coverage, with a slight preference for target undercoverage over spillage to normal tissue (Figure 3C). Overall, all 15 plans met the prespecified target-coverage criteria, supporting dosimetric plan-generation feasibility; however, five plans exceeded at least one bowel objective.

## 4. Discussion

The ability to generate treatment plans meeting the prespecified target-coverage criteria in all 15 cases supports the dosimetric plan generation feasibility of HFRT with a central SIB boost in selected patients with large, bulky RPS. Ten plans met both target coverage criteria and all prespecified OAR objectives, whereas five met the target-coverage criteria but exceeded at least one bowel objective. These findings do not establish the clinical deliverability, safety, or efficacy of this treatment approach. While this cohort consisted of patients who ultimately underwent surgical resection, these cases were selected for dosimetric analysis because of their large tumor size, complex anatomic distribution, and proximity to and involvement of organs at risk, features that can also present substantial treatment-planning challenges in patients with unresectable disease. These patients underwent treatment at a high-volume sarcoma center, and some may not have been considered surgical candidates in other clinical settings [22,23]. Nevertheless, this cohort should not be considered equivalent to a population with unresectable RPS. Unresectability may specifically result from involvement or encasement of critical structures, including bowel or major vessels, and these anatomic relationships may create substantially different dosimetric limitations. Prescription doses were selected using EQD2 with an α/β ratio of 3 to account for the low α/β typically seen in soft tissue sarcomas and ensure sufficient dose to tumor; however, the true α/β for retroperitoneal sarcomas remains uncertain. Our cohort was also liposarcoma-enriched, with 12 of 15 patients (80%) having either well-differentiated or dedifferentiated liposarcoma and the remaining three histologies each represented by a single patient. The reported dosimetric feasibility therefore principally reflects the growth patterns and anatomic behavior of retroperitoneal liposarcoma. This histologic composition also bears on the biological assumptions underlying the prescription, as the α/β ratio may vary across soft tissue sarcoma subtypes; the α/β of 3 Gy applied uniformly here is a modeling assumption rather than a subtype-specific value.

Our approach to prioritizing OAR constraints over target allowed maintenance of target coverage while generally achieving the selected OAR planning objectives, although in some patients bowel metrics remained limiting. In regard to target coverage, HI and CI were used to assess plan quality. The HI for iGTV (0.21) and SIB (0.05) were both well within the recommended value of 0–0.5, where a score closer to zero indicates higher homogeneity and thus better plan quality [24]. The SIB HI values show particularly good homogeneity, with the slightly higher iGTV HI likely due to dose fall-off from the SIB rather than from particular hot or cold spots. In terms of PTV CI, a value of one indicates ideal conformation, while a value less than one is indicative of partial undercoverage of treatment volume, and a value greater than one is indicative of dose spillage to healthy tissues [25]. A score between 0.9 and 1, as achieved in our cohort, is considered to be an acceptable minor difference and is acceptable to allow for sparing of normal tissue. Using an SIB offers distinct advantages over a traditional sequential boost that may be relevant to the treatment of RPS. From a logistical perspective, the SIB approach permits differential dose escalation within a single treatment course. Tumor necrosis and immunomodulatory effects have been proposed as potential biological mechanisms supporting central tumor dose escalation, although their relevance to this regimen and disease setting remains unknown [17]. However, whether central dose escalation or use of an SIB translates into improved tumor control or other clinical benefits in RPS cannot be determined from the present dosimetric study.

Both bilateral and unilateral contralateral kidney constraints were obtained for all patients with two kidneys that were both discernible from the tumor; however, for many of the patients in our cohort, the ipsilateral kidney was either directly abutting or within the treatment field, which is common in RPS. Since our cohort comes from a pre-operative radiation patient population, all patients with two kidneys had undergone prior renal scan and were confirmed to have adequate contralateral kidney function for ipsilateral nephrectomy. Per consensus guidelines, when RPS involves or closely abuts a kidney to the extent that nephrectomy is considered necessary, and renal scan conveys sufficient contralateral kidney function, then RT treatment planning should focus on maximal sparing of the contralateral kidney [21]. Thus, in this cohort, where all patients had received renal scans showing sufficient contralateral kidney function, the unilateral contralateral kidney constraint and target coverage were prioritized as a hard constraint over the bilateral kidney constraint, which was used as a soft constraint. This clinical context differs importantly from that of patients with unresectable disease, in whom nephrectomy is not planned, and preservation of functional renal tissue in both kidneys may be clinically important. Therefore, the kidney prioritization strategy used in this preoperative cohort should not be directly extrapolated to all patients with unresectable RPS. Among the 11 patients with reported bilateral kidney metrics, four exceeded the bilateral kidney planning constraint, although the contralateral kidney metric remained below the specified constraint in these cases. These findings suggest that bilateral renal dose may represent an additional limitation of this treatment approach when applied to patients in whom preservation of both kidneys is required. Application to unresectable disease would therefore require individualized consideration of tumor-kidney relationships, baseline renal function, and the relative function of each kidney. However, in patients who are deemed unresectable for other reasons unrelated to the kidney, a renal scan prior to radiation planning may still be beneficial to determine if there can be laxity introduced to bilateral constraint in favor of reaching better target coverage with a hard contralateral kidney constraint.

In this study, we used loop-based bowel contours and constraints. Loop-based contouring enables accurate assessment of dose to visible bowel segments adjacent to high-dose target volumes, which is particularly important for minimizing the risk of gastrointestinal toxicity in intensity-modulated or highly conformal treatments [26]. Compared to the bowel bag, which represents a potential space rather than actual bowel anatomy, loops provide more anatomically specific dosimetric estimates near targets, especially when high-quality simulation imaging is available and when considering dose escalation [27,28]. Additionally, there is evidence that exceeding bowel bag dose does not correlate with acute gastrointestinal toxicity in patients receiving pre-operative radiation for retroperitoneal sarcoma [27]. Analysis of contour agreement among radiation oncologists experienced in treating sarcoma showed similar agreement in contouring of both bowel bag and small bowel, indicating both approaches are reproducible [28]. Although bowel motion introduces some variability, studies suggest that contouring loops near the planning target volume (PTV) captures clinically significant dose regions more accurately than relying on the bowel bag, which may overestimate dose exposure and lacks anatomical specificity [29]. In a study of prostate cancer patients, the bowel contouring approach was associated with no difference in planned dose or bowel toxicity [30]. Importantly, however, the choice of contouring approach does not eliminate uncertainty introduced by bowel motion during a multi-fraction treatment course. Bowel dose in the present study was assessed using bowel anatomy from a single simulation dataset. Bowel loops are inherently mobile, and their position relative to the high-dose target may change between treatment fractions. Consequently, dosimetric evaluation based on the simulation scan cannot ensure that bowel sparing would be maintained throughout a 15-fraction treatment course. This consideration is particularly important with a central SIB to 52.5 Gy, for which movement of bowel into or toward the high-dose region could result in greater delivered bowel dose than predicted by the original treatment plan. Although loop-based contouring provides anatomically specific information at the time of simulation, it does not account for this interfractional variation. Accordingly, this single-scan analysis cannot establish cumulative delivered bowel dose or confirm that the planned bowel constraints would be maintained throughout treatment.

Meeting bowel dose constraints is a well-recognized challenge in the treatment of retroperitoneal sarcoma (RPS) because of the large size and anatomic location of these tumors. Accordingly, it is unsurprising that proximity to the bowel represented the most challenging organ at risk (OAR) in our cohort and is likely to remain a major dosimetric limitation in patients with unresectable disease. In our cohort, 5 of 15 patients (33%) slightly exceeded at least one bowel metric. In each case, the metric was exceeded by less than 1% of the specified constraint and was in the large and/or small bowel, but not the duodenum. For all these patients, while the maximum dose was slightly above the constraint, the D0.03 constraint was met, indicating that the slight overdose was likely a point dose less than 0.03 cc. Additionally, the one patient that exceeded large bowel V42.75 did so by only 0.1 cc. Despite the recognized difficulty in meeting bowel constraints, it appears that patients being treated for RPS have lower significant acute GI toxicity compared to gastrointestinal or gynecologic cancer patients [27]. A recent study has shown that exceeding historical constraints when treating abdominopelvic sarcomas may increase the incidence of grade 1–2 acute GI toxicities during RT; however, long-term toxicities remained rare [31]. Additionally, bowel constraints for hypofractionated regimens are still under study, with multiple studies showing tolerable effects at variable doses [32,33]. These data do not establish that exceeding bowel objectives is clinically acceptable. Rather, the observed deviations identify bowel as the principal limitation of the approach and support prospective evaluation with careful image guidance, predefined dose-modification rules, and toxicity monitoring. Given the difficulty in meeting bowel constraints and the uncertainty introduced by interfraction bowel motion, potential clinical implementation would require careful image guidance and assessment of bowel position throughout treatment. Daily volumetric image guidance may allow identification of clinically meaningful changes in bowel position relative to the high-dose target, and adaptive replanning or other mitigation strategies may be necessary when bowel moves into a high-dose region. However, the ability of these strategies to maintain bowel constraints throughout treatment would require prospective evaluation using cumulative delivered-dose assessment.

## 5. Conclusions

This study is dosimetric in nature, and therefore does not provide clinical outcome data. Although the ability to meet the prespecified target-coverage criteria in all 15 plans supports dosimetric plan-generation feasibility, the study does not establish clinical deliverability, toxicity, clinical safety, or efficacy. In addition, most patients in the cohort ultimately proceeded to surgical resection, suggesting that they may have had more favorable anatomy and tumors than the intended target population of unresectable cases; this may limit the applicability of our findings to the broader unresectable cohort. However, the tumors represented in our study were of large size and located throughout the abdomen and pelvis. The purpose of this study was to evaluate dosimetric plan-generation feasibility, not demonstrate superiority over conventional fractionation. We did not perform a formal geometric analysis of tumor-to-bowel distance or bowel volume overlapping the high-dose region. Such an analysis could help identify which anatomic configurations are most amenable to this technique and is a natural extension for future work, particularly given that bowel proximity was the dominant dosimetric constraint in this cohort. Finally, despite being relatively robust for a study in this rare disease, the sample size remains modest and may restrict the generalizability of the results. Prospective clinical evaluation would be required to determine the deliverability, safety, toxicity, local control, and quality-of-life outcomes associated with this approach in patients with unresectable RPS.

## Figures and Tables

**Figure 1:**
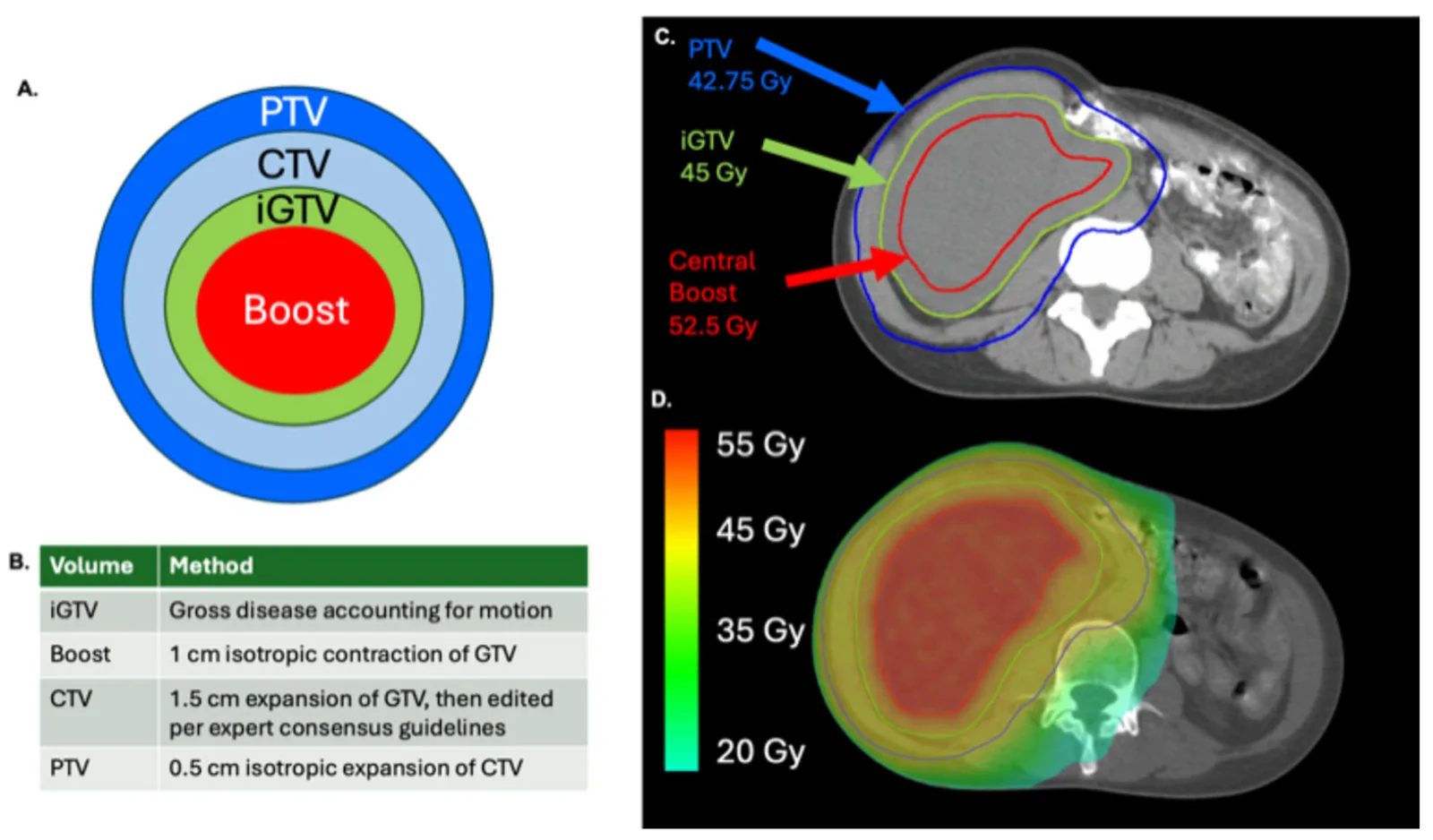
Radiation therapy treatment plan. (**A**). Diagram of approach to boost, iGTV, CTV, and PTV relative to each other. (**B**). Method of obtaining iGTV, boost, CTV, and PTV. (**C**). Example of contouring with prescription dose on a sample patient. (**D**). Example of final treatment plan on sample patient.

**Figure 2:**
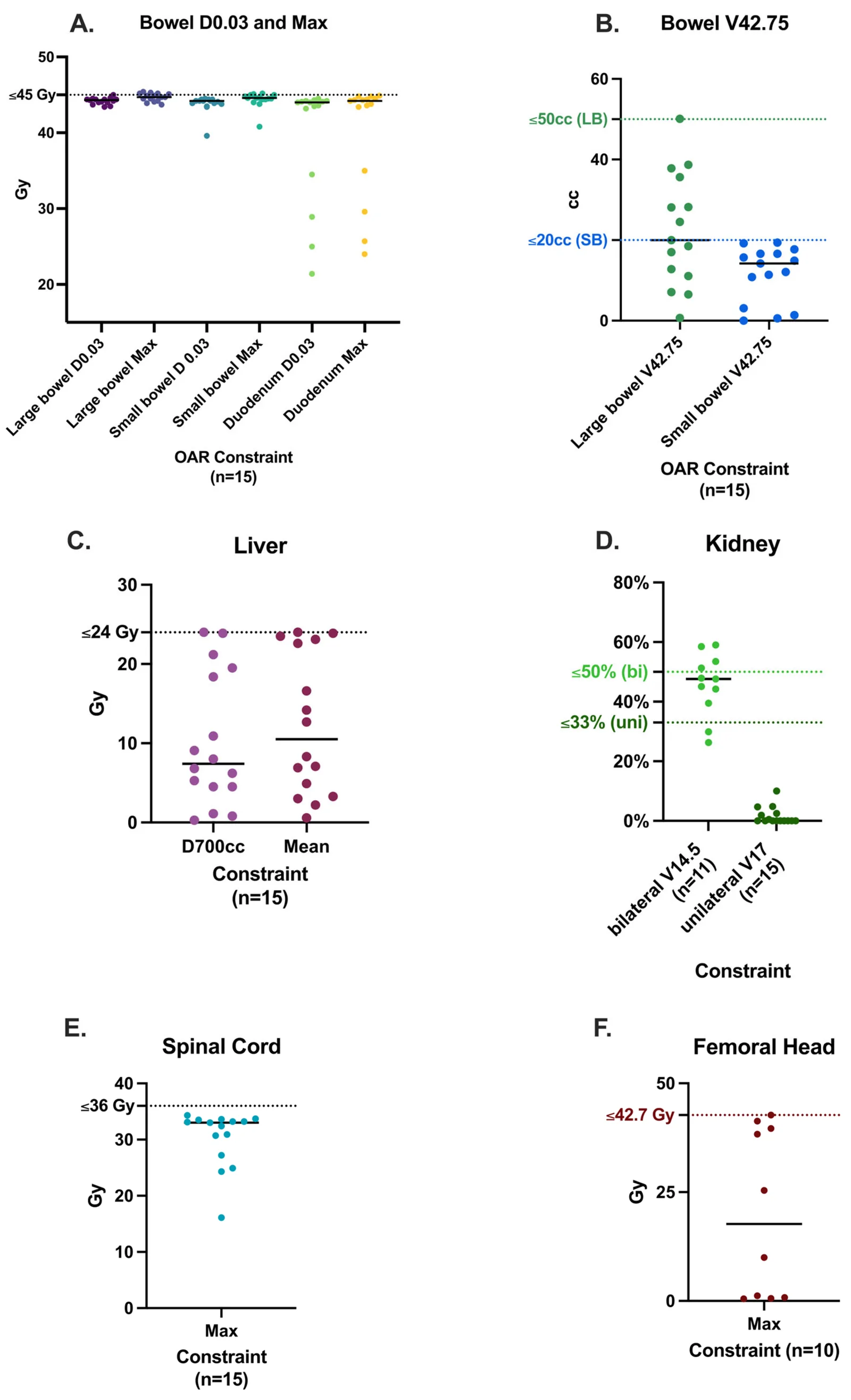
OAR constraints. Representation of OAR metrics. Each dot is a single patient; the straight line is the mean metric per constraint across all patients, and the dotted line is the constraint limit. (**A**). Bowel D0.03 and maximum dose for large bowel, small bowel, and duodenum, all with constraint <45 Gy, n = 15 for all metrics. (**B**). V42.75 dose for large bowel and small bowel with constraints <50 cc and <20 cc, respectively; n = 15 for both metrics. (**C**). Liver D700 cc and mean liver dose, both constraints < 24 Gy, n = 15 for both. (**D**). Bilateral kidney V14.5 dose and unilateral contralateral kidney V17 dose, constraints < 50% and <33%, respectively; n = 11 and n = 15, respectively. (**E**). Maximum spinal cord dose with constraint <36 Gy, n = 15. (**F**). Maximum femoral head dose with constraint <42.7 Gy, n = 10.

**Figure 3:**
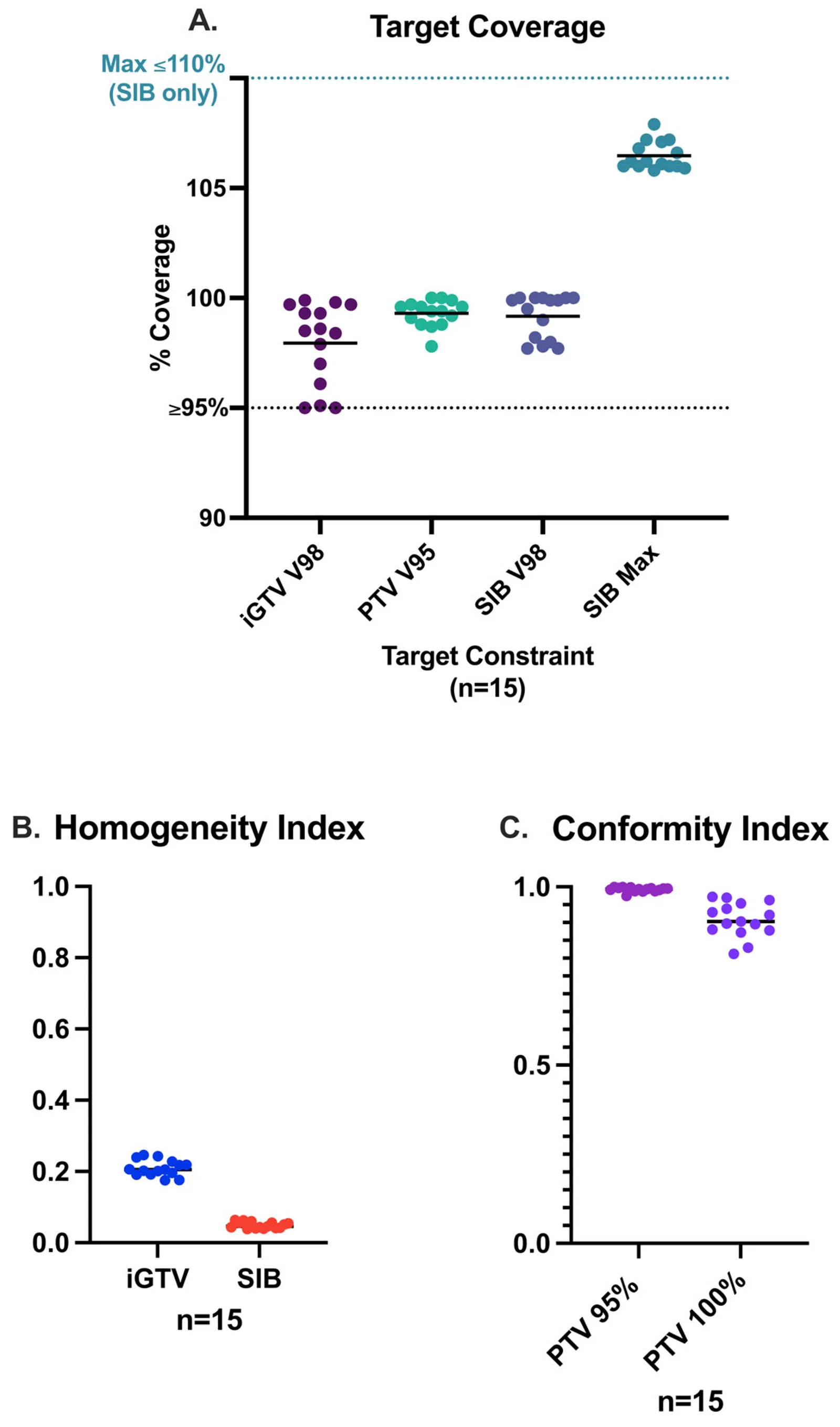
Target coverage. Representation of target volume dose. Each dot is a single patient; the straight line is the mean metric per target across all patients, and the dotted line is the target goal, n = 15 for all metrics. (**A**). Percentage coverage of iGTV V98, PTV V95, and SIB V98, all with a goal of ≥95% coverage; SIB maximum dose with a goal of <110%. (**B**). Homogeneity Index for iGTV and SIB. (**C**). Conformity Index for PTV using 95% and 100% isodose lines.

**Table 1:** Organs At Risk (OAR) metrics.

OAR	Constraint
Spinal Canal	Max 36 Gy
Small Bowel	Max * 45 Gy; V42.75 < 20 cc
Large Bowel	Max * 45 Gy; V42.75 < 50 cc
Duodenum	Max * 45 Gy
Kidneys (bilateral) **	V14.5 Gy < 50%
Kidney (unilateral)	V17 Gy < 33%
Bladder	V42.75 Gy < 50%; V30 Gy < 60%
Liver	Mean < 24 Gy; 700 cc < 24 Gy
Femoral heads	Max 42.7 Gy; V38 < 50%
Anus, vulva, and/or perineum	V22.5 Gy < 50%

* Both point max and D0.03 max recorded for bowel structures; otherwise point max used; ** Bilateral kidney constraint recorded in all patients with two kidneys but only used as hard constraints in patients whose tumors did not involve the ipsilateral kidney. Note: OAR constraints for hypofractionated approach were selected based on national and international guidelines, discussion with sarcoma and gastrointestinal radiation oncology experts, and Equivalent Dose in 2 Gy fractions (EQD2) calculations. Constraint-specific sources, whether each represents a hard limit or planning goal, and EQD2 equivalents are provided in Table S5.

**Table 2:** Prescription dose levels for simultaneous integrated boost.

Target	Dose	EQD2 α/β = 3	EQD2 α/β = 5	EQD2 α/β = 10	Constraint
PTV	2.85 Gy × 15 fractions (42.75 Gy)	50 Gy	47.9 Gy	45.8 Gy	≥95% of PTV at 95% of prescription dose
iGTV	3 Gy × 15 fractions (45 Gy)	54 Gy	51.4 Gy	48.8 Gy	≥95% of iGTV at 98% of prescription dose
SIB Central Boost (1 cm contraction)	3.5 Gy × 15 fractions (52.5 Gy)	68.2 Gy	63.8 Gy	59.1 Gy	≥95% of SIB at 98% prescription dose

**Table 3:** Patient characteristics.

Characteristic	n (%)
Gender	
Female	10 (66.7%)
Male	5 (33.3%)
Histology	
WDLPS	4 (26.7%)
DDLPS	8 (53.3%)
Undifferentiated Pleomorphic Sarcoma	1 (6.7%)
Pleomorphic Spindle Cell Sarcoma	1 (6.7%)
Myxoid and Spindle Cell Sarcoma	1 (6.7%)
Grade	
Low	6 (40.0%)
Intermediate	7 (46.7%)
High	2 (13.3%)
Stage	
cT2N0M0	4 (26.7%)
cT3N0M0	4 (26.7%)
cT4N0M0	7 (46.7%)
Tumor location	
Retroperitoneal	14 (93.3%)
Pelvis	1 (6.7%)
Tumor Size	
Mean (range)	18.3 cm (5.8–28.2 cm)
Median (interquartile range)	17.1 cm (14.1–25.5 cm)
Side	
Right	8 (53.3%)
Left	7 (46.7%)
Recurrent	
Yes	3 (20.0%)
No	12 (80.0%)
Ipsilateral Nephrectomy	
Prior to radiation therapy	2 (13.3%)
After radiation therapy	11 (73.3%)
Partial after radiation therapy	1 (6.7%)
No	1 (6.7%)

## Data Availability

Research data are stored in an institutional repository and will be shared upon request to the corresponding author.

## Supplementary Materials

The following supporting information can be downloaded at: https://www.mdpi.com/article/10.3390/cancers18193114/s1, Table S1. Summary of Prior Literature; Table S2. Target Constraints and Homogeneity/Conformality Statistics per Patient; Table S3. Bowel Constraints Per Patient; Table S4. Non-Bowel OAR Constraints Per Patient; Table S5. EQD2 Conversion of 15-Fraction OAR Dose Constraints.
