# Supplementary material for: Dosimetric Feasibility of Hypofractionated Radiation Therapy with Simultaneously Integrated Central Boost for Retroperitoneal Sarcoma
Source: Cancers (Basel). 2026 Sep 25;18(19):3114. doi: 10.3390/cancers18193114 (PMC13664376; doi:10.3390/cancers18193114)
Supplement: Supplementary file 1 [file cancers-18-03114-s001.zip › cancers-4497667-supplementary.pdf]

Table S1. Summary of Prior Literature

Study	n	Study type	Dose	Results
**Lee et al. Adv in Rad Oncol, 2020**	1	Case Report of Unresectable RPS	PTV 36 Gy in 25 fractions, simultaneous central boost: 20 Gy (4Gy/fx once weekly)	Well-tolerated, stable disease for 10 months
**Boyce-Fappiano et al. Adv in Rad Oncol, 2022**	18	Retrospective analysis of unresectable or metastatic RPS	PTV: 45Gy in 15 fractions simultaneous central boost to GTV or CTV	1 year LC 73%, median systemic therapy break of 13 months
**Liveringhouse et al. IJROBP, 2023**	69 standard 34 SIB	Retrospective analysis of resectable nonmetastatic RPS	SIB pts received 2.05-2.3Gy per fraction, median dose for SIB group was 45Gy + 12.5Gy (total 57.5Gy)	SIB associated with improved abdominopelvic control and recurrence free survival without a detriment in toxicity
**Medici et al. Radiol Case Rep 2023**	1	Case Report: Bulky RP liposarcoma	20Gy in 4 fractions twice daily to macroscopic tumor, 40Gy in 4 fractions twice daily to central part of tumor	Complete metabolic response throughout entire tumor mass on PET. Patient succumbed to metastatic spread to bone, liver and lung after 9 months but no local disease progression.
**Yu et al. Radiotherapy & Oncology, 2024**	18	Retrospective analysis of bulky (>5cm) unresectable sarcomas	20Gy in 5 fractions with central boost to 50Gy	Well-tolerated with symptom improvement, 1-year OS 61%, Freedom from local progression 83.3%, freedom from distant progression 34.8%
**Wong et al. Radiation Oncology Journal, 2025**	8	Phase I of RPS	25Gy in 5 fractions starting dose, escalation stage to 30Gy in 5 fractions followed by surgery	Safe, intra- and peri-operatively
**Burner et al Adv in Rad Oncol, 2025**	5	Case series of unresectable RPS	On average doses of 25-45Gy delivered to outer part of tumor w/ central boost to 56-75Gy (total of 25 fractions in each case, in 3 patients cone down, in 2 patients SIB)	No nephropathy, treatment tolerated well

Table S2. Target Constraints and Homogeneity/Conformality Statistics per Patient

Patient	plan type	iGTV V98	SIB V98	SIB Max	PTV V95	HI iGTV	HI SIB	CI PTV 100%	CI PTV 95%
1	VMAT	98.5	99.9	107.1	97.8	0.19	0.06	0.90	0.97
2	IMRT	95.1	99.5	106.6	99.6	0.24	0.05	0.90	1.00
3	VMAT	95	97.8	107.2	98.8	0.25	0.06	0.83	0.99
4	IMRT	98.6	100	106.2	99.7	0.19	0.04	0.97	1.00
5	IMRT	96.1	100	106	99.6	0.23	0.04	0.81	1.00
6	VMAT	97	98.2	106	99.4	0.21	0.06	0.88	0.99
7	VMAT	95	100	106.2	99.2	0.24	0.04	0.95	0.99
8	VMAT	99.9	99.9	105.9	99.9	0.18	0.04	0.96	1.00
9	VMAT	99.7	98	106.1	99.6	0.20	0.05	0.94	1.00
10	VMAT	98.4	100	105.8	100	0.22	0.04	0.87	1.00
11	VMAT	99.8	100	106	99.4	0.20	0.04	0.93	0.99
12	VMAT	99.3	99.9	106	99.1	0.21	0.04	0.90	0.99
13	VMAT	99.3	97.7	106.8	98.8	0.20	0.06	0.88	0.99
14	VMAT	97.9	97.7	107.9	98.7	0.22	0.06	0.92	0.99
15	IMRT	99.7	99	107.2	100	0.18	0.05	0.97	1.00

Table S3. Bowel Constraints Per Patient

Patient	Large Bowel V42.75	Large bowel D0.03 Max	Large Bowel Point Max	Small bowel V42.75	Small bowel D0.03 Max	Small bowel Point Max	Duodenum D0.03 Max	Duodenum Point Max
1	35.6	44.5	45.1	17.7	44.2	45.1	34.5	35
2	50.1	45	45.4	1.4	43.9	44.6	43.5	43.6
3	17	44.4	44.7	3.1	43.8	44.4	44.2	44.5
4	11.1	44.2	44.5	0	39.6	40.8	44.1	44.3
5	0.7	43.4	43.9	0.6	43.5	44.5	43.2	43.4
6	24.5	44.4	45.3	14.2	44	44.4	28.9	29.6
7	28.1	44.3	44.9	16.6	44.4	44.9	21.4	24
8	6.5	44	44.2	14.9	44.1	44.4	44.1	44.4
9	7.1	43.7	44.1	15.7	44.2	44.6	44	44.3
10	28.2	44.4	44.9	19.4	44.4	44.8	44.3	44.9
11	18.5	44.1	44.7	10.8	44.2	44.6	25	25.7
12	20	44	44.5	11.4	43.9	45	43.6	43.8
13	37.8	44.4	45.2	12.1	44.2	44.5	44.2	44.8
14	38.7	44.6	45.2	16.6	44.5	45	44.5	44.8
15	12.8	43.5	43.7	19.2	43.4	43.8	44	44.2

Table S4. Non-Bowel OAR Constraints Per Patient

Patient	Liver D700cc	Liver Mean	Spinal Cord Point Max	kidney bilateral v14.5	kidney unilateral V17	Femoral head Max
**1**	8	8.3	33.5	59	4.7	0.8
**2**	18.4	23.9	32.4	53.5	4.8	38.3
**3**	10.9	12.7	33.6	58.5	2.5	39.6
**4**	19.5	22.6	33.1	26.3	1.9	
**5**	1.1	3	30.7	N/A*	0	N/A**
**6**	4.5	4.9	32.7	N/A*	0	N/A**
**7**	5.3	7.1	33.2	44.2	0	N/A**
**8**	6.8	23.5	27.2	N/A*	10	0.5
**9**	23.9	23.1	33.2	45.1	0	42.7
**10**	0.3	0.6	16.1	29.9	0	0.6
**11**	0.8	2.2	24.3	39.5	0	N/A**
**12**	4.5	3.3	24.9	N/A*	0	41.3
**13**	6.2	6.9	30.9	47.6	0	25.4
**14**	9.1	14.2	34.3	47.9	0	1.2
**15**	21.2	16.6	33.7	51.3	0.5	10

*Bilateral kidney dose not applicable because patient has single kidney

**Femoral head dose not applicable because tumor was sufficiently superior that femoral heads were not included in field of view for planning CT

Table S5. EQD2 Conversion of 15-Fraction OAR Dose Constraints

OAR	15-fx constraint	d (Gy/fx)	α/β used	EQD2
**Spinal canal**	Max 36 Gy	2.4	2	39.6 Gy
**Small bowel**	Max 45 Gy	3.0	3	54.0 Gy
**Small bowel**	V42.75 < 20 cc	2.85	3	50.0 Gy
**Large bowel**	Max 45 Gy	3.0	3	54.0 Gy
**Large bowel**	V42.75 < 50 cc	2.85	3	50.0 Gy
**Duodenum**	Max 45 Gy	3.0	3	54.0 Gy
**Kidneys (bilateral)**	V14.5 < 50%	0.97	2	10.8 Gy
**Kidney (unilateral)**	V17 < 33%	1.13	2	13.3 Gy
**Bladder**	V42.75 < 50%	2.85	3	50.0 Gy
**Bladder**	V30 < 60%	2.0	3	30.0 Gy
**Liver**	Mean < 24 Gy	1.6	3	22.1 Gy
**Liver**	D700cc < 24 Gy	1.6	3	22.1 Gy
**Femoral heads**	Max 42.7 Gy	2.85	3	49.9 Gy
**Femoral heads**	V38 < 50%	2.53	3	42.1 Gy
**Anus/vulva/perineum**	V22.5 < 50%	1.5	3	20.3 Gy

Note: EQD2 conversion of partial-volume constraints is approximate, since the LQ model converts dose, not the volume threshold attached to it. Spinal canal, liver, duodenum, and unilateral-kidney constraints were prioritized as hard limits during planning. Small- and large-bowel constraints were prespecified high-priority planning objectives; observed deviations were reported separately and were not reclassified as compliant. Bilateral-kidney, femoral-head, bladder, and anus/vulva/perineum constraints were treated as soft planning objectives. Five of 15 patients exceeded at least one bowel objective by less than 1% where bowel directly abutted the tumor (see Results 3.2).
